# Proposed follow up programme after curative resection for lower third oesophageal cancer

**DOI:** 10.1186/1477-7819-8-75

**Published:** 2010-09-04

**Authors:** LH Moyes, JE Anderson, MJ Forshaw

**Affiliations:** 1 Oesophagogastric Unit University Department of Surgery Glasgow Royal Infirmary 84 Castle Street Glasgow G4 0SF, UK

## Abstract

The incidence of oesophageal adenocarcinoma has risen throughout the Western world over the last three decades. The prognosis remains poor as many patients are elderly and present with advanced disease. Those patients who are suitable for resection remain at high risk of disease recurrence. It is important that cancer patients take part in a follow up protocol to detect disease recurrence, offer psychological support, manage nutritional disorders and facilitate audit of surgical outcomes. Despite the recognition that regular postoperative follow up plays a key role in ongoing care of cancer patients, there is little consensus on the nature of the process. This paper reviews the published literature to determine the optimal timing and type of patient follow up for those after curative oesophageal resection.

## Introduction

The incidence of adenocarcinoma of the oesophagus and gastric cardia has been increasing throughout the Western world over the last three decades while the incidence of squamous cell carcinoma has remained stable [1]. The prognosis is poor as many patients are elderly and present with advanced disease making them unsuitable for curative resection. The five-year survival for all patients is less than 15% [2].

Surgery remains the primary curative treatment for oesophageal adenocarcinoma, providing permanent relief of dysphagia and offering the possibility of cure. A recent compilation of the world literature by Jamieson et al reported overall five-year survival rates in Western countries of up to 30% in those undergoing curative resection [2]. These results represent an improvement from previous decades and can be attributed to preoperative staging investigations, better assessment of operative risk improved operative techniques and better perioperative management. Today in most large European oesophagogastric units mortality rates are less than 5% [3,4]. The National Oesophagogastric Cancer Audit in England and Wales covering 93% of all resectional units recently reported an in-hospital mortality rate of 5.0% following oesophagectomy [5]. Reductions in postoperative mortality, whilst possibly improving one year survival rates, do not translate into an improvement for long term survival as many patients continue to present with recurrent disease following apparently curative surgery. A study by Mariette showed that almost 50% of recurrent disease develops within two years and this is within a group which has been highly selected as resectable [6]. Studies have shown that T stage, N status, lymph node ratio and completeness of resection (R status) are important prognostic factors in survival post oesophagectomy [7-11].

In order to provide the best quality of care to patients who have undergone curative resection, clinical follow up is recommended. This allows the clinician to detect and treat benign complications of their treatment, detect recurrent or metastatic disease, assess and manage nutritional disorders, provide psychosocial support to patients and their families and facilitate auditing of surgical outcomes [12].

Despite the recognition that regular postoperative follow up plays a key role in ongoing care of cancer patients, there is little consensus on the nature of the process.

This paper aims to review the published literature to determine the optimal timing and type of patient follow up and the nature of investigations to be performed.

## Literature Search

A literature search was performed using Medline, Embase and Cochrane databases searching for English literature available since 1975. The search was performed with MESH terms "oesophageal cancer", "adenocarcinoma of gastro-oesophageal junction", "follow up guidelines", "disease recurrence", "clinical practice guidelines", "prognosis" and "survival". All related articles were examined. Where evidence was lacking for oesophagogastric cancer follow up, the literature on other common cancers such as breast and colon was examined.

## Results

Despite the recognition that regular postoperative follow up plays a key role in the ongoing care of cancer patients, there is little consensus on the nature of the process and this is reflected in the lack of scientific data in the literature. There are no randomised controlled trials or other studies or series assessing the appropriate follow up protocol for patients after curative oesophagogastric resection.

### Published guidelines

There are four sets of guidelines available in the literature concerning the follow up of patients after curative oesophagogastric resection - the British Society of Gastroenterology 2002, the Scottish Intercollegiate Guidelines Network (SIGN) 2006, the European Society of Medical Oncology 2009 and the National Comprehensive Cancer Network (NCCN) 2009 [12-15]. As stated, there are no randomised controlled trials which specifically investigate the duration and frequency of follow up. In all cases, adenocarcinoma of the distal oesophagus and the gastroesophageal junction are regarded as similar. A recent study has shown there is no difference in surgical management, survival or recurrence between these two tumours so their follow up should be regarded as the same [16].

The main points from the guidelines demonstrate a lack of defined or standardised follow up protocols. All the guidelines admit that routine follow up does not impact on patient survival outcomes but should be used to concentrate on palliating symptoms from benign complications of treatment, nutritional status and psychosocial support. Studies have shown that cancer patients prefer regular follow up for ongoing support and reassurance and as a point of contact should new symptoms or concerns arise [14,17,18]. It is important to differentiate between those patients who are essentially asymptomatic or have minor chronic symptoms in whom a routine follow up approach is appropriate, and those patients developing new or suspicious symptoms in whom more urgent assessment and investigation is required.

The following points will be briefly discussed: the personnel involved in follow up protocols, the investigations performed, the cost effectiveness of intensive programmes, the effect of follow up on quality of life and the treatment options available when recurrence occurs.

### Personnel involved in follow up

The guidelines suggest that follow up should be coordinated within a multidisciplinary team involving the patient, surgeon, oncologist, radiation oncologist, specialist nurse and dietician. The BSG guidelines suggest that clinical nurse specialists could have a developed role in the routine review of cancer patients, thus reducing the need for medical based reviews in those patients who are well. This would allow medical staff to focus on those who need further input or investigation [12]. This system is used successfully in follow up programmes of other cancers [19,20].

Many patients prefer continued care in the primary care setting, avoiding the stress of hospital visits, so routine follow up visits could be shared between the hospital clinic and the primary care setting. General practitioners embarking on follow up will be only those willing to undertake the work and there must be good and rapid communication lines between the surgical team and the primary care physician.

### Frequency of follow up visits

There is little consensus in the literature regarding the frequency of follow up visits. Many of the follow up protocols used in the literature are fairly rigorous and are often part of clinical trials looking at postoperative outcomes such as disease recurrence and survival in patients undergoing potentially curative resection.

The literature search was expanded to include trials concerning follow up of common cancers such as breast, lung and colorectal. There are several randomised controlled trials examining the effects of intensive versus non intensive follow up for these common cancers. The American Society of Clinical Oncology breast cancer follow up guidelines have shown no significant survival advantage with an intensive surveillance protocol instead of a clinically based protocol [21]. There is no consensus regarding the follow up of patients after curative resection for colorectal cancer. The British Society of Gastroenterology guidelines suggest there is no evidence that frequent follow up visits carry significant survival advantages or benefits to patients whereas other meta-analyses have shown some survival benefit as metachronous tumours can be diagnosed earlier [22,23]. However these tumours have completely different biology and treatment options compared with oesophagogastric tumours, so recommendations regarding the follow up of these cancers while providing a useful framework may not be transferable to oesophagogastric cancer patients.

The recurrence rate after oesophagogastric resection is high (34-79%) with more than half recurring within the first year and most presenting within two years of surgery [24-26]. Mariette showed that 45.7% patients developed recurrence within 12 months of the operation, and the time from recurrence to death was 7 months [6]. De Manzoni confirmed similar results with 80% patients undergoing potentially curative resection developing recurrence within 24 months [7]. The depth of tumour invasion (T stage) and number of positive lymph nodes (N stage) are the main factors predictive of recurrent disease after R0 resections [11,26]. The literature suggests that patients with lymphovascular invasion, positive resection margins and high lymph node ratio have poorer outcomes, but at present there is no evidence that these factors are used in any follow up protocol or to focus follow up [27-29].

Based on these patterns of recurrence, many units recommend an initial postoperative visit four to six weeks after surgery to check on wounds, nutritional status and record any early complications from treatment. Thereafter most centres vary between additional visits at three to four month intervals for the first year, six monthly reviews the second year, and annually thereafter [6-8,30-32]. Patients tend to be followed up for five years but there is no evidence supporting this besides the fact that most recurrences occur within this time.

### Investigations performed

The SIGN guidelines state "no evidence has been identified to support regular imaging or measurement of serum tumour markers in the follow up of patients with gastroesophageal cancer outside clinical trials" [13]. However there are numerous different imaging protocols used throughout Europe and North America.

Many patients have routine serum biochemistry performed at each visit, and some centres measure carcinoembryonic antigen (CEA) as a tumour marker which can rise in the presence of recurrent disease. This is not universal and studies have shown that CEA for oesophageal adenocarcinoma is not reliable [33] with sensitivity rates for detecting recurrence between 19- 39% and specificity rates of 89%.

The investigations used by most centres for routine follow up are gastroscopy and biopsy, USS of neck, computed tomography (CT), positron emission tomography (PET), combined CT-PET and CT/US guided biopsy of any identified suspicious lesion. There is no evidence to suggest endoscopic ultrasound should be used for routine follow up, as CT provides comparable results for assessing local recurrence [34,35]. Some units perform an annual CT of the neck, chest and abdomen and a gastroscopy although some prefer six monthly intervals [6,7,16,31]. Other groups prefer investigation only if there is a clinical indication as treatment options for recurrent disease are limited and the prognosis poor [30,32].

Combination PET-CT is now increasingly being used to detect recurrent disease following surgical resection [34,36]. PET-CT appears to be both sensitive and specific for diagnosing distant metastases and local recurrence [37]. PET-CT may be useful when there is diagnostic doubt after other investigations. At present there is little evidence in the literature to suggest that PET-CT should be involved in routine follow up protocols. There is some evidence that unsuspected recurrence can be identified in asymptomatic patients but it difficult to be sure this is in the patients' best interests.

### Quality of Life

The argument against intensive follow up is that it may not lead to true survival advantage but is purely due to lead time bias. Earlier diagnosis therefore not only fails to prolong life but may reduce the quality of life due to increased anxiety resulting from earlier knowledge of an inevitable death [38]. Clinicians' concerns regarding quality of life are often based on survival whereas patients tend to be more concerned about fears of disability and financial or social consequences. Follow up visits and additional investigations often cause temporary anxiety in 30% patients, although this is then replaced with reassurance and optimism in the case of negative results [39]. Most patients value follow up as good news can improve their quality of life, but even in cases of recurrent disease where there are often no treatment options, patients appreciate the honesty and time given to be with family and deal with their affairs [40].

Meta-analyses and randomised controlled trials in breast and colorectal cancer patients have identified no difference between quality of life and emotional well being in those involved in either an intensive or non intensive follow up programme [41].

### Cost effectiveness

There have been no trials assessing the cost effectiveness of intensive follow up of patients after oesophagogastric resection as there have been with other common cancers [23,41]. It is therefore difficult to present scientific data supporting an intensive follow up programme with investigation for asymptomatic patients to detect disease recurrence particularly when there is no evidence that earlier detection affects outcome. Intensive follow up programmes with annual endoscopy and CT in asymptomatic patients, with further investigations if required are costly to the health service and do not impact on patient survival and often cause anxiety while awaiting the results. It seems sensible that investigations are performed in symptomatic patients so that treatment of benign complications or palliative therapy for recurrent disease can be commenced. However routine investigation of an asymptomatic population in whom there are rarely any curative treatment options available for recurrent disease cannot be recommended.

### Recurrence following curative surgery

Cancer recurrence is a common problem after oesophagectomy for oesophageal cancer. The mode of recurrence is often classified into three patterns: local recurrence occurs at the anastomosis, regional recurrence in the mediastinum or upper abdomen at the site of previous oesophageal resection, and distant recurrence defined as disease in other organs or peritoneum [6,7]. Most recurrences occur within two years of surgery. The median survival rate following regional recurrence was 7 months while patients with local or distant disease have survival rates of only 5 months.

For many years there has been debate surrounding the most appropriate resection for adenocarcinomas of the mid and lower oesophagus. Most studies suggest that while there may be a modest early survival advantage in those undergoing a transhiatal approach, the long term survival advantage does not seem to be affected according to approach [42-45]. One study did report lower recurrence rates and improved survival with the transhiatal approach but this has not been supported by other studies [46]. A recent follow up study assessing five year survival rates in a large cohort of cancer patients undergoing transhiatal and transthoracic oesophagectomy concluded no difference in recurrence rates or overall survival between the two groups. However they suggest a transthoracic approach may be more appropriate in those with between one and eight positive nodes as this offers a 41% increase in five year survival [47].

### Treatment of recurrent disease

Patients presenting with disease recurrence may be asymptomatic, the recurrent disease being identified on routine surveillance, or symptomatic with dysphagia, pain, weight loss or early satiety. Treatment options for locally recurrent oesophageal cancer are limited. Reoperation for resection of locally recurrent oesophageal disease is technically challenging as the approach is made more difficult by the presence of scar tissue, and the operative field has probably been previously irradiated. A study by Schipper et al analysed the outcome of 23 patients who underwent re-resection of locally recurrent disease [48]. Approximately one third were found to have unresectable disease intraoperatively. Two, three and five year survival rates for patients with R0 re-resections were 62%, 44% and 35% respectively. The authors conclude that re-resection of locally advanced disease is associated with considerable morbidity (59% complication rate and 7% operative mortality) but long term survival is possible in those undergoing complete re-resection. However this is only possible in a small minority of carefully selected patients.

Therefore, for the majority of patients palliative therapies become the mainstay of treatment, ensuring the best quality of life and symptom control. Palliative therapies for recurrent disease include radiation therapy, chemotherapy, endoscopic interventions and a combination of these. Endoscopic stenting remains the most popular method for alleviation of dysphagia although other endoscopic treatments such as laser treatment, photodynamic therapy and argon plasma coagulation can be used. These treatments are useful for smaller areas of recurrence and provide good symptomatic relief, although repeated treatments are often required to maintain symptom control. To date the optimal endoscopic intervention for palliation of dysphagia has not been established with a systematic review showing no difference between the various endoscopic therapies [49]. The choice of endoscopic treatment will depend upon the endoscopist's experience, the individual patient and their pathology and the available resources in the endoscopy unit.

Radiation treatment, either external beam radiotherapy or intraluminal brachytherapy, allows high dose of radiation to be delivered directly to the luminal surface of the tumour. Studies have shown that brachytherapy is as effective as stenting for the palliation of dysphagia.

Chemotherapy remains an option for the palliation of recurrent and metastatic oesophagogastric cancer, although there is limited evidence regarding its benefit. It is often used in conjunction with other therapies, particularly radiotherapy. Although there may be a survival advantage associated with chemotherapy, patients need to attend regular hospital visits and the regimens can be associated with significant side effects. These factors may reduce the quality of the time patients have left [50].

## Discussion

The ideal follow up strategy should offer regular clinical visits and where appropriate, focused investigation to detect disease recurrence and offer support, but not so frequently as to cause undue anxiety and harming their quality of life.

The goals of patient follow up are to treat benign complications which have arisen from surgery such as anastomotic strictures and delayed gastric emptying requiring endoscopic dilatation which tend to present within the first year following surgery [51]. All patients should undergo nutritional assessment before and after surgery and a dietician must be involved in each patient's care. Weight loss is a concerning symptom but immediately following surgery this may be simply due to inadequate caloric intake and adaptation of eating habits. A further goal of follow up is to offer and maintain psychosocial support. Some patients will need more support than others and the clinical nurse specialist plays an invaluable role in this area. Follow up plays a role in identifying disease recurrence as early as possible to ensure patients receive the appropriate palliation or further treatment if indicated. Finally it is imperative that physicians record their morbidity, recurrence and survival rates so we can continually endeavour to audit and improve the management of these patients.

We would suggest the following as a possible follow up strategy, as illustrated in Table 1, for clinically well patients undergoing curative oesophagogastric resection based on current practice, guidelines and typical peaks of recurrence. This is by no means wholly evidence based, but a pragmatic approach must be adopted in order to provide patients with the reassurance and support they require, while being able to investigate and manage symptoms as they present in order to ensure the best quality of life for patients. Any symptomatic patient needs directed investigations, most units starting with either gastroscopy or CT neck, chest and abdomen.

**Table 1:** Summary of recommendations

Visit	Postoperative Timing	Purpose
1	4 weeks	• Post operative wound check
		• Assessment of nutritional status
		• Discussion of pathology results
		• Referral for further treatment

2	3 months	• Nutritional assessment
		• Identification of benign complications

3	6 months	• As above

4	9 months	• As above

5	12 months	• As above

6	18 months	• As above

7	24 months	• Final assessment of general health
		• If remains well, discharge back to GP
		• Open access to surgical/oncology team if any new concerning symptoms

8	36 months	• Hospital clinic visit (or GP if preferred)

9	48 months	• Hospital clinic visit (or GP if preferred)

10	60 months	• Hospital clinic visit (or GP if preferred)
	Any time	• Development of new symptoms require
		○ Assessment
		○ Investigation - Initial CT chest/abdomen/pelvis and endoscopy with further investigation as clinically indicated (PET-CT, bone scan, US)
		○ Palliative therapies and care team

Patients should undergo an initial postoperative check within four weeks of discharge to rule out any immediate wound complications, discuss pathology results and any further treatment if required, to identify nutritional problems and discuss any unresolved issues. The rest of the follow up should be focused on nutritional and psychosocial support and identifying any concerning symptoms - all of these issues tending to arise within the first two years after resection. Any patient developing recurrence can have their symptoms palliated efficiently and appropriate support offered.

Patients should be assessed clinically with a history and physical examination at three monthly intervals in the first year and six monthly in the second year. An annual clinical review should be performed for years three, four and five years at which point the patient is discharged back to the primary care team. The NCCN guidelines which are widely used in the US advocate a similar hospital based follow up programme. For some patients who are highly motivated, and have a willing primary care team, there may be a role of annual follow up under the care of their general practitioner after year 2 with back to the surgical or oncological team if any new issues arise. However we suspect the majority of general practitioners, surgeons or patients may prefer follow up under the surgeons who know the specifics about their operative history and therefore hospital follow up would be appropriate. Where the follow up visit is in hospital, there should be a multidisciplinary approach to avoid duplication of examinations and investigations, and inconvenience to patients [12]. Clinical investigations should only be performed if any specific new symptom develops.

### Prediction of disease recurrence

As over 50% patients develop some form of recurrence, predicting which patients are most likely to recur would be of value. This clearly would have most impact in the preoperative period ensuring appropriate en bloc resection and adjuvant therapy to reduce the risk of recurrence. At present, intensive postoperative follow up strategies that pick up earlier asymptomatic recurrence have no impact on overall survival - so it may not be worth the cost and effort, and potential upset to the patient. The TNM staging system is currently used in assessing patients preoperatively and planning their management. However a recent revision to the TNM staging classification for oesophageal adenocarcinoma has shown a new N classification based on the number and location of the involved lymph nodes improves the prognostic power [52]. The revised classification of nodes (N0, none: N1, one to five: N2, six or more) was prognostically significant when applied to 313 patients undergoing oesophagectomy with curative intent. The revised classification seems to stage individual patients more accurately and may alter postoperative treatment regimens and follow up.

A group from the Netherlands have developed a prognostic normogram to aid prediction of disease specific survival after oesophagectomy which seems to be superior to TNM staging. However at present the normogram estimates prognosis only after resection but the authors feel it may be clinically helpful in providing more reliable prognostic information and in time tailor follow up protocols [53].

There is much in the literature over the past few years concerning the role of the systemic inflammatory response and its relationship to cancer outcomes. Studies have shown that those patients with increased pre-treatment levels of C-reactive protein are more likely to have poorer survival [54]. A Japanese group suggest that those with increased CRP levels are at higher risk of disease recurrence, while Deans and colleagues have suggested that CRP incorporated into a clinical prognostic scoring system may aid the MDT decision making process [55,56]. The benefit of this scoring system over conventional pathological factors is that the four variables (clinical stage, performance score, weight loss and serum CRP concentration) can be used prospectively to guide decisions at the time of initial diagnosis, and give realistic prognostic information. This could stratify patients into high risk groups, requiring closer follow up.

Artificial neural networks, the combination of molecular markers and tumour and patient factors, can predict outcomes from oesophageal cancer. A preliminary model incorporating 199 variables in more than 400 oesophageal cancer patients has been developed which may be more suitable than the TNM staging system in classifying an individual's patient recurrence risk and survival [57].

## Conclusions

There is no consensus in the literature regarding the frequency, duration and imaging modalities used in the follow up of patients undergoing curative resection for oesophageal cancer. The clinical importance of follow up for detection of recurrence at an early stage is unclear, because there is no evidence that early detection of recurrence results in good treatment outcome.

The aim of follow up should be to provide patients with the best quality of life by providing reassurance and dealing with new symptoms as they arise. Over investigation causes anxiety and we suggest appropriate investigations should be used at the discretion of the clinician when new symptoms arise. Based on the limited evidence currently available, a five year hospital based clinical follow up programme for patients undergoing curative oesophagogastric resection with transfer back into the primary care sector seems a reasonable strategy for patients who are well. However there may be some patients who would prefer annual follow up for years 3-5 under the care of their general practitioner, which may be feasible provided rapid communication links are open between primary and secondary healthcare systems. Urgent referral is required for any patient developing new or worrisome symptoms. New strategies, clinical prognostic scoring models and tumour markers may be of benefit in the future.

## Competing interests

The authors declare that they have no competing interests.

## Authors' contributions

JA and LM performed the literature search and LM and MF wrote the manuscript. All authors read and approved the final manuscript.
